# Parasites, pathogens and commensals in the “low-impact” non-native amphipod host *Gammarus roeselii*

**DOI:** 10.1186/s13071-017-2108-6

**Published:** 2017-04-20

**Authors:** Jamie Bojko, Karolina Bącela-Spychalska, Paul D. Stebbing, Alison M. Dunn, Michał Grabowski, Michał Rachalewski, Grant D. Stentiford

**Affiliations:** 1 Faculty of Biological Sciences, University of Leeds, Leeds, LS2 9JT UK; 2 Pathology and Molecular Systematics Team, Centre for Environment, Fisheries and Aquaculture Science (Cefas), Weymouth Laboratory, Weymouth, Dorset DT4 8UB UK; 3 Department of Invertebrate Zoology & Hydrobiology, University of Lodz, Banacha 12/16, 90-237 Lodz, Poland; 4 Epidemiology and Risk Team, Centre for Environment, Fisheries and Aquaculture Science (Cefas), Weymouth Laboratory, Weymouth, Dorset DT4 8UB UK; 5 European Union Reference Laboratory for Crustacean Diseases, Centre for Environment, Fisheries and Aquaculture Science (Cefas), Weymouth Laboratory, Weymouth, Dorset DT4 8UB UK

**Keywords:** Microsporidia, *Cucumispora*, Parasite, Amphipoda, Invasive, Virus, Wildlife disease

## Abstract

**Background:**

Whilst vastly understudied, pathogens of non-native species (NNS) are increasingly recognised as important threats to native wildlife. This study builds upon recent recommendations for improved screening for pathogens in NNS by focusing on populations of *Gammarus roeselii* in Chojna, north-western Poland. At this location, and in other parts of continental Europe, *G. roeselii* is considered a well-established and relatively ‘low-impact’ invader, with little understanding about its underlying pathogen profile and even less on potential spill-over of these pathogens to native species.

**Results:**

Using a combination of histological, ultrastructural and phylogenetic approaches, we define a pathogen profile for non-native populations of *G. roeselii* in Poland. This profile comprised acanthocephalans (*Polymorphus minutus* Goese, 1782 and *Pomphorhynchus* sp.), digenean trematodes, commensal rotifers, commensal and parasitic ciliated protists, gregarines, microsporidia, a putative rickettsia-like organism, filamentous bacteria and two viral pathogens, the majority of which are previously unknown to science. To demonstrate potential for such pathogenic risks to be characterised from a taxonomic perspective, one of the pathogens, a novel microsporidian, is described based upon its pathology, developmental cycle and SSU rRNA gene phylogeny. The novel microsporidian *Cucumispora roeselii* n. sp. displayed closest morphological and phylogenetic similarity to two previously described taxa, *Cucumispora dikerogammari* (Ovcharenko & Kurandina, 1987), and *Cucumispora ornata* Bojko, Dunn, Stebbing, Ross, Kerr & Stentiford, 2015.

**Conclusions:**

In addition to our discovery extending the host range for the genus *Cucumispora* Ovcharenko, Bacela, Wilkinson, Ironside, Rigaud & Wattier, 2010 outside of the amphipod host genus *Dikerogammarus* Stebbing, we reveal significant potential for the co-transfer of (previously unknown) pathogens alongside this host when invading novel locations. This study highlights the importance of pre-invasion screening of low-impact NNS and, provides a means to document and potentially mitigate the additional risks posed by previously unknown pathogens.

**Electronic supplementary material:**

The online version of this article (doi:10.1186/s13071-017-2108-6) contains supplementary material, which is available to authorized users.

## Background

Understanding and interpreting the role played by pathogens in the invasion mechanisms of their hosts is becoming increasingly important as legislative pressure is placed upon managers to prevent and control wildlife disease [1, 2]. Often, the pathogens of invasive hosts are little known or cryptic, requiring dedicated screening efforts to elucidate underlying parasites and pathogens that may be vectored to new habitats by non-native species (NNS) [2, 3].

The Amphipoda constitute a diverse crustacean group with many species displaying invasive characteristics that have spread throughout Europe *via* invasion corridors [4]. Poland is considered part of one such invasion corridor connecting the Ponto-Caspian region to western Europe [4, 5], making it an important study site for both recipient and donor populations of amphipods destined to reach other parts of Europe. Most non-native amphipod taxa found in Poland originate from the Ponto-Caspian region; however some exceptions exist. One example includes *Gammarus roeselii* Linnaeus of Balkan origin and documented to have invaded western Europe (including Poland, Italy, France and Germany) over a century ago, with a relatively low impact [6–10]. This species continues to extend its non-native range, now encompassing the Apennine Peninsula [11]. Although the host *per se* is considered a low impact NNS [12], current risk assessments associated with its spread do not take account of its underlying pathogen profile, nor the effect of these pathogens on receiving hosts and habitats. Several parasites and pathogens of *Gammarus roeselii* are known, including the acanthocephalans *Polymorphus minutus* (Zeder, 1800) [13]; *Pomphorhynchus laevis* (Zoega in Müller, 1776) [14] and *Pomphorhynchus tereticollis* (Rudolphi, 1809) [15]; and the microsporidians *Dictyocoela muelleri* Terry, Smith, Sharpe, Rigaud, Timothy & Littlewood, 2004 (unofficial genus) [16]; *Dictyocoela roeselii* Terry, Smith, Sharpe, Rigaud, Timothy & Littlewood, 2004 (unofficial genus) [16]; *Nosema granulosis* Terry, Smith, Bouchon, Rigaud, Duncanson, Sharpe & Dunn, 1999 [16]; and several *Microsporidium* spp*.* [17, 18] (see Table 1).

**Table 1:** Parasites and pathogens associated with *Gammarus roeselii* and available reference for each association

Parasite taxon	Species	Location	Available data	Reference
Acanthocephala	*Polymorphus minutus*	France	Visual	[13]
	*Pomphorhynchus tereticollis*	Denmark	DNA sequence and visual	[15]
	*Pomphorhynchus laevis*	France	Visual	[14]
Microsporidia	*Dictyocoela muelleri*	France	DNA sequence	[16]
	*Dictyocoela roeselii*	France	DNA sequence	[16]
	*Nosema granulosis*	France	DNA sequence	[16]
	*Microsporidium* sp. G	Germany	DNA sequence	[17]
	*Microsporidium* sp. 505	Germany	DNA sequence	[17]
	*Microsporidium* sp. nov. RR2	Germany	DNA sequence	[17]
	*Microsporidium* sp. nov. RR1	Germany	DNA sequence	[17]
	*Microsporidium* sp. group F	Germany	DNA sequence	[18]
	*Microsporidium* sp. group E	Germany	DNA sequence	[18]
	*Microsporidium* sp. 2	Germany	DNA sequence	[18]

Acanthocephalan parasites have been observed to cause various behavioural [14], physiological [19] and biochemical changes [20] on their amphipod host, which could alter their host’s invasive capability. Some of the microsporidians infecting *G. roeselii* (Table 1) are taxa previously associated with other invasive amphipod hosts [17, 21, 22]. Some unassigned ‘*Microsporidium*’ spp*.* infecting *G. roeselii* may in fact reside within the genus *Cucumispora* Ovcharenko, Bacela, Wilkinson, Ironside, Rigaud & Wattier, 2010 [23]. This genus currently contains two species isolated from invasive amphipods: *Cucumispora dikerogammari* (Ovcharenko & Kurandina, 1987) and *Cucumispora ornata* Bojko, Dunn, Stebbing, Ross, Kerr & Stentiford, 2015. Like their hosts, existing members of the genus *Cucumispora* may also be of Ponto-Caspian origin due to their identification within tissues of *Dikerogammarus* spp*.* native to that region [23]. However, the detection of *Cucumispora-*like sequences (based upon PCR diagnostics and sequencing) in non-native *G. roeselii* originating from the Balkans, suggests that microsporidia belonging to the *Cucumispora* may have a range extending further than the Ponto-Caspian region depending on whether *G. roeselii* is a co-evolved host [17]. *Cucumispora* spp*.* have been associated with a variable host range, inferring there is a possibility for transmission from Ponto-Caspian invaders; concluding that *Cucumispora* spp. are likely emerging diseases among amphipods [24].

In order to understand the pathogen profile of a low-impact non-native species and assess the risk of pathogen introduction from such an invader, we surveyed a population of *G. roeselii* in north-western Poland with an aim to understand which pathogen groups were present, whether the pathogen profile of a low-impact invader was different from that of high-impact invaders, and whether these pathogens pose a significant threat to native wildlife. We present the outcome of this survey here as the first comprehensive pathogen survey of *G. roeselii*. Using a combination of field sampling, histology, transmission electron microscopy and molecular diagnostics, we define an array of novel pathogens associated with this host and taxonomically define a new member of the microsporidian genus *Cucumispora* infecting *G. roeselii*. We discuss these results relative to the impact of these pathogens on population success and impact in Poland, their potential risk of transfer with further spread of this host across Europe and the importance of screening low-impact, NNS for pathogens without simply focussing on screening high-impact invasive hosts.

## Methods

### Collection, dissection and fixation of *Gammarus roeselii*

*Gammarus roeselii* were sampled using standard hydrobiological nets and kick-sampling from the banks of a stream in Chojna, north-western Poland (Oder river catchment) (N52.966, E14.42906) on 23/06/2015. A total of 156 specimens were collected: 8 were fully dissected to remove muscle and hepatopancreas to fix for histology (Davidson’s freshwater fixative), transmission electron microscopy (TEM) (2.5% glutaraldehyde) and molecular diagnostics (96% ethanol), and 148 were injected on site with fixative for histological screening. Carcasses in fixative, or live animals, were transported to Łόdź University, Poland for storage and/or dissection.

### Histopathology and transmission electron microscopy

Specimens preserved in Davidson’s freshwater fixative were transferred to 70% methylated spirit after 24–48 h and infiltrated with paraffin wax using an automated tissue processor (Peloris, Leica Microsystems, Milton Keynes, UK). Wax embedded tissues were then sectioned sagittally a single time on a Finesse E/NE rotary microtome (Thermofisher, Hemel Hempstead, UK) (3–4 μm thickness). Sections were glass mounted and stained using haematoxylin and alcoholic eosin (H&E) and examined using a Nikon Eclipse E800 light microscope. Images were captured using an integrated LEICA™ (Leica, Milton Keynes, UK) camera.

Sample preparation and observation *via* transmission electron microscopy (TEM) for muscle and hepatopancreas tissues dissected from *G. roeselii* followed that used by Bojko et al. [22].

### Molecular diagnostics

Muscle tissue dissected from a single infected *G. roeselii* was confirmed positive for microsporidiosis *via* visual, histological and TEM diagnostics. Muscle tissue from the same individual was fixed in ethanol upon dissection and used for DNA extraction. DNA extraction was performed using a standard phenol-chloroform method. SSU rRNA gene amplification was performed using the primers MF1 (5′-CCG GAG AGG GAG CCT GAG A-3′) and MR1 (5′-GAC GGG CGG TGT GTA CAA A-3′) [25] and 2.5 μl of DNA template (~30 ng/μl) in a GoTaq flexi PCR reaction (per reaction: 10 pM of each primer; 0.25 M of each dNTP; 1.25 U Taq Polymerase; 2.5 mM MgCl_2_) in a total volume of 50 μl. T_c_ settings were: 94 °C (5 min), 94 °C - 60 °C - 72 °C (each 1 min; 35 cycles), 72 °C (10 min). Amplicons were observed using gel electrophoresis on a 2% agarose gel (30 min/120 V) producing a microsporidian band at ~800 bp. This band was excised and purified for forward and reverse sequencing *via* Eurofins genomics barcode-based sequencing service (Eurofinsgenomics, UK).

### Phylogenetics and sequence analysis

The final SSU rRNA gene sequence for this microsporidian was 825 bp sequence length, which was placed into BLASTn (NCBI) to retrieve identical or close hits. The sequence was placed alongside several SSU rRNA gene sequences used by Ovcharenko et al. [23] to form the initial description of *Cucumispora dikerogammari* (GQ246188.1), as well as some closely linked, recently described microsporidian sequences [*C. ornata* (KR190602.1); *Paradoxium irvingi* Stentiford, Ross, Kerr, Bass & Bateman, 2015 (KU163282.1); *Hyperspora aquatica* Stentiford, Ramilo, Abollo, Kerr, Bateman, Feist, Bass & Villalba, 2016 (KX364284.1), *Unikaryon legeri* (Dollfus, 1912) (KX364285.1)], and all available partial or complete sequences from BLAST that link with close similarity to *C. dikerogammari* (GQ246188.1) and could potentially be candidates for the genus *Cucumispora*.

The sequences were aligned with MAFFT 7.017 [26] using default values, in Geneious 6.1.8 [27]. The phylogeny reconstruction was performed in MEGA 7 [28] using the maximum-likelihood [29] and Neighbour-Joining [30] methods. Clade credibility was assessed using bootstrap tests with 1,000 replicates [31]. The T92 model of evolution with gamma-distributed rate heterogeneity (G) was selected for the dataset using the complete deletion model selection algorithm implemented in MEGA 7. Clade IV microsporidian species were used as the outgroup to root the tree.

## Results

### Histological observations

**Fig. 1:**
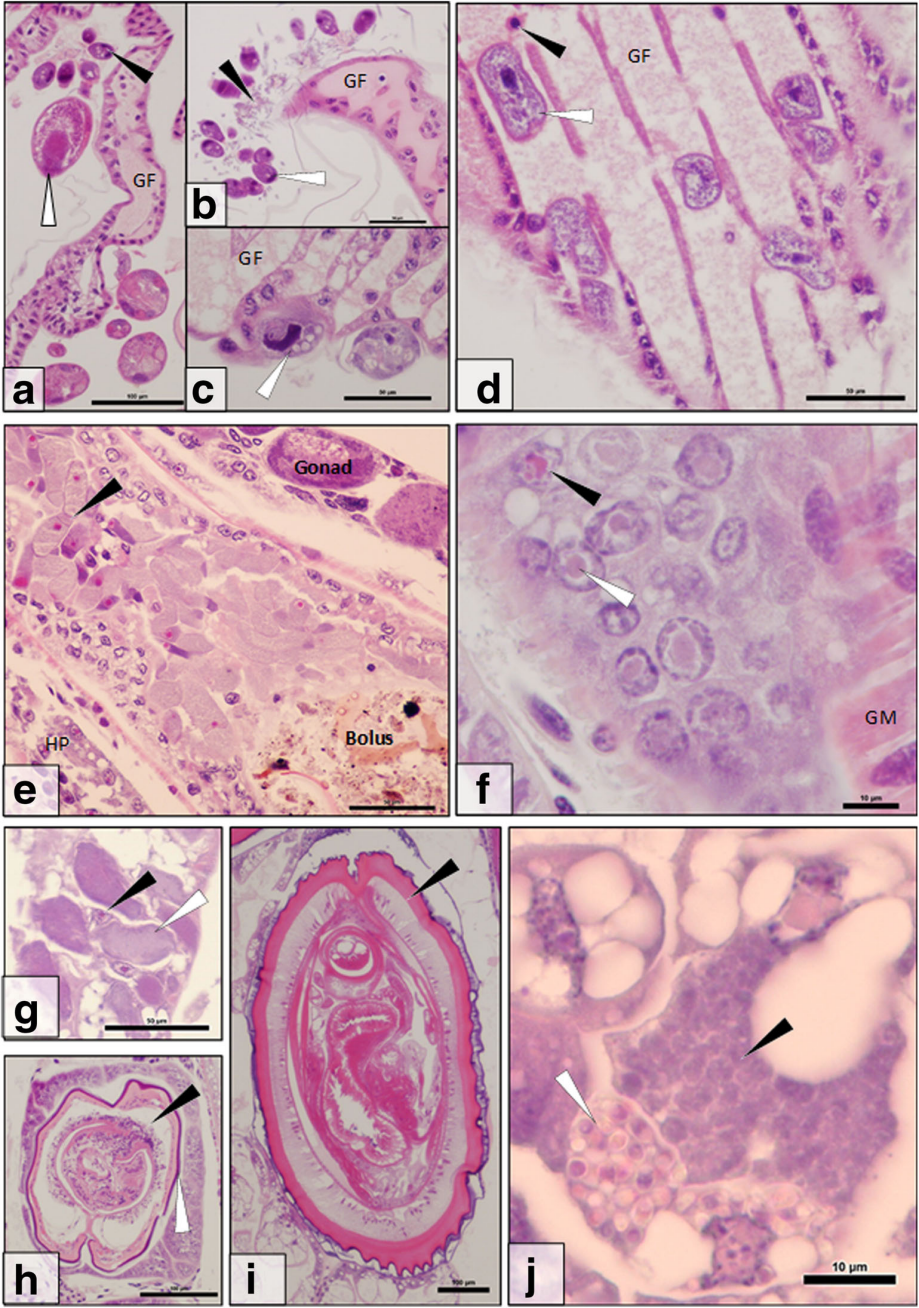
Parasites and pathogens observed during the histological screen of *Gammarus roeselii*. **a** External rotifers (*white arrow*) and stalked ciliated protists (*black arrow*) clustered around a gill filament (GF). **b** Externally associated ciliated protists (*white arrow*) and filamentous bacteria (*black arrow*) clustered around a gill filament (GF). **c** Ciliated protists (*white arrow*) embedded into the gill filament (GF). **d** Ciliated protists (*white arrow*) present in the blood stream (blood cell = *black arrow*) of the gill filament (GF). **e** Dense cluster of gregarines (*black arrow*) in the gut alongside bolus, gonad and hepatopancreas (HP). **f** Putative nuclei-targeted gut epithelia virus displaying nuclear hypertrophy due to expanding viroplasm (*arrows*) (GM = gut muscle). **g** Putative rickettsia-like organism in the cytoplasm of hepatopancreatocytes (*white arrow*). The nucleus (*black arrow*) is unaffected. **h** Digenean trematode (black arrow), present with external pearling (*white arrow*), encysted internally within *G. roeselii*. **i** *Polymorphus* sp*.* encysted internally within *G. roeselii*. **j** An unidentified microsporidian pathogen in the cytoplasm of infected hepatopancreatocytes. Developing (*black arrow*) and mature spore stages (*white arrow*) of the pathogen can be clearly identified in separate cells. *Scale-bars*: **a**, **h**, **i**, 100 μm; **b**-**e**, **g**, 50 μm; **f**, **j**, 10 μm

Overall, 156 *G. roeselii* specimens from Chojna were histologically screened, revealing several parasites, pathogens and commensals. Altogether, 14 associations were catalogued. These included: epibiotic stalked ciliated protists (Fig. 1a, b); epibiotic, gill-embedded ciliated protists (Fig. 1c); epibiotic filamentous bacteria (Fig. 1b); epibiotic rotifers (Fig. 1a); a parasitic peritrichioius protist (Fig. 1d); gut-dwelling gregarines (Fig. 1e); a putative gut virus (Fig. 1f); a putative rickettsia-like organism (RLO) in the hepatopancreas (Fig. 1g); digenean trematodes (Fig. 1h ); acanthocephalans, including: *Polymorphus minutus* (Fig. 1i) and *Pomphorhynchus* sp*.* (no image); a microsporidian restricted to the hepatopancreas (Fig. 1j); a bacilliform virus from the nuclei of the hepatopancreas with confirmed morphological information and a muscle-targeting microsporidian, which is also taxonomically identified herein using histology, TEM and phylogenetic analysis. Prevalence information for all parasites and pathogens is contained in Table 2.

**Fig. 2:**
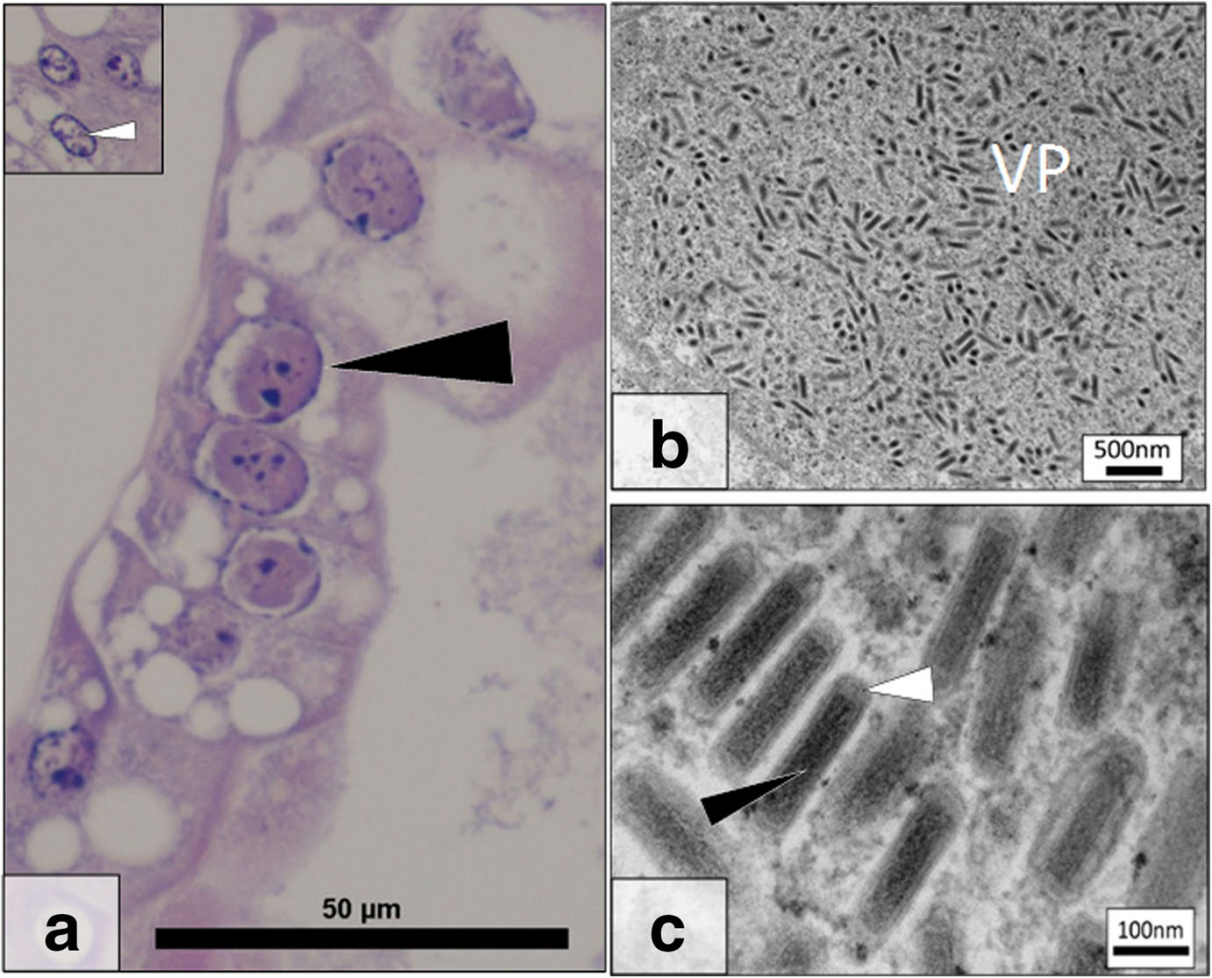
*Gammarus roeselii Bacilliform Virus* (GrBV) histopathology and ultrastructure. **a** Several virally infected, hypertrophic, nuclei (*black arrow*) in the hepatopancreas. Inset at the same magnification details a cluster of uninfected nuclei (*white arrow*). **b** Electron micrograph detailing a growing viroplasm (VP) in a nucleus of the hepatopancreas. **c** High magnification image of the bacilliform virus present with electron dense core (*black arrow*) and membrane (*white arrow*) in a paracrystalline array within a heavily infected cell nucleus. *Scale-bars*: **a**, 50 μm; **b**, 500 nm; **c**, 100 nm

**Fig. 3:**
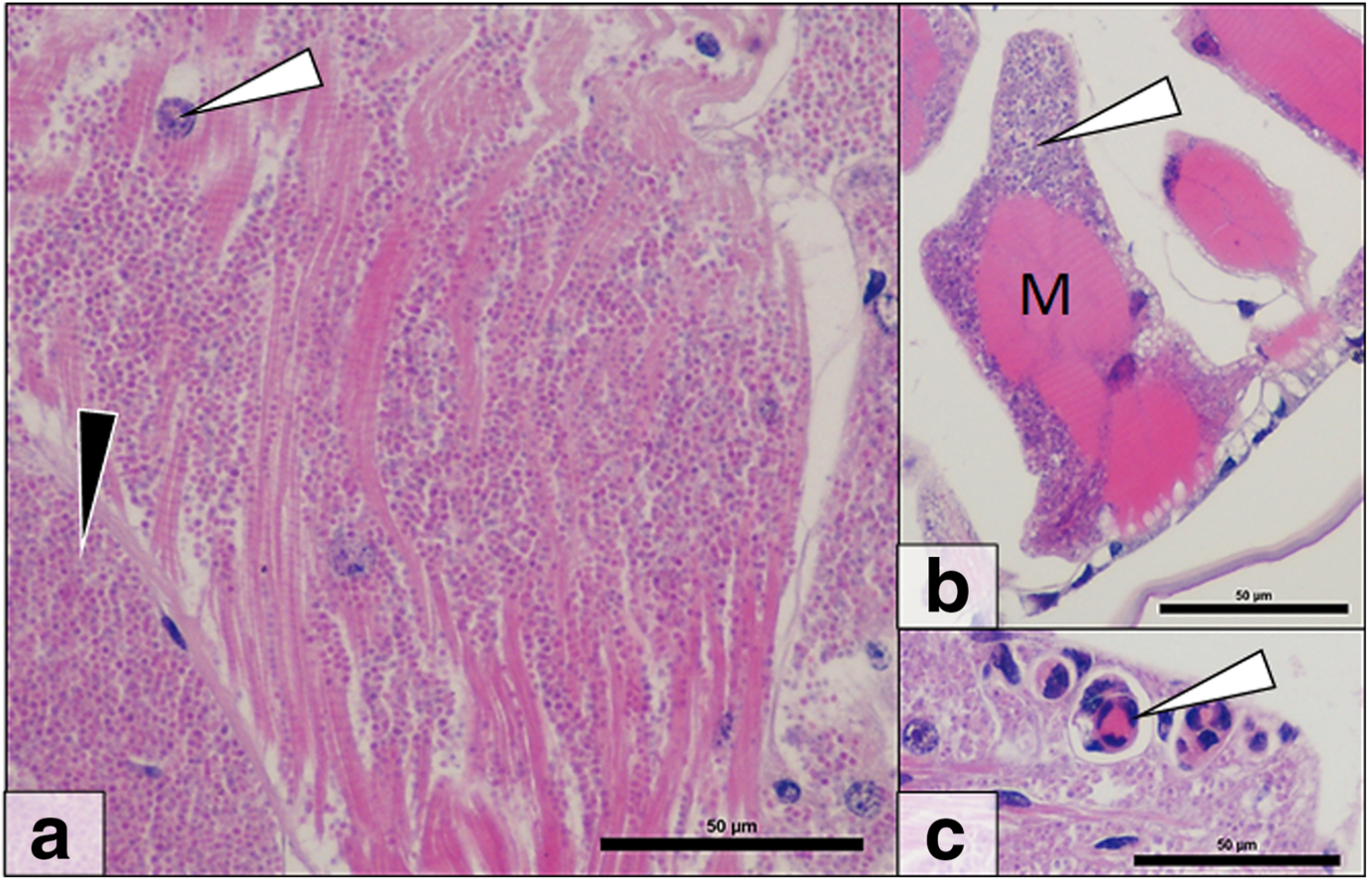
*Cucumispora roeselii* n. sp. histopathology. **a** Microsporidian spores (*black arrow*) can be seen throughout the musculature in heavy infections. Muscle nuclei (*white arrow*) can be seen amongst parasite spores. **b** Early stage microsporidian infected muscle blocks (M) demonstrate initial sarcolemma infection (*white arrow*). **c** Immune reactions (*white arrow*) towards microsporidian infection. *Scale-bars*: 50 μm

**Fig. 4:**
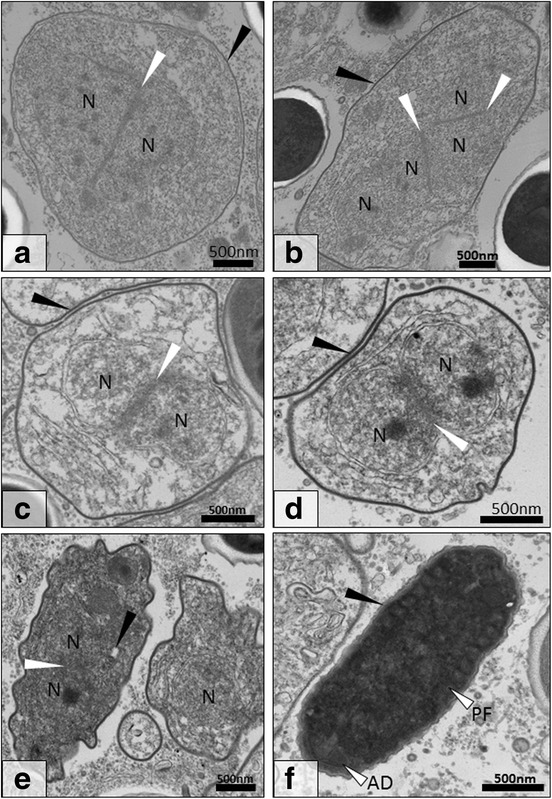
Transmission electron micrograph of early spore development for *Cucumispora roeselii* n. sp. **a** Diplokaryotic meront displaying attached nuclei (N; *white arrow*). Note the thin cell membrane (*black arrow*). **b** Tetranucleate cell displaying four attached nuclei (N; *white arrows*) with a thickening cell wall (*black arrow*). **c** After division, two early diplokaryotic (N; *white arrow*) sporoblasts are produced with further cell membrane thickening (*black arrow*). **d** Early diplokaryotic (N; *white arrow*) sporoblast displaying further thickening of the cell membrane (black arrow). **e** The early sporoblast begins to become electron dense and condense with some early development of spore organelles such as the polar filament (*black arrow*). **f** Fully condensed sporoblast development stage present with electron dense cytoplasm and coiled polar filament (PF) and anchoring disk (AD). At this stage the formation of the early endospore is visible (*white arrow*). *Scale-bars*: 500 nm

**Fig. 5:**
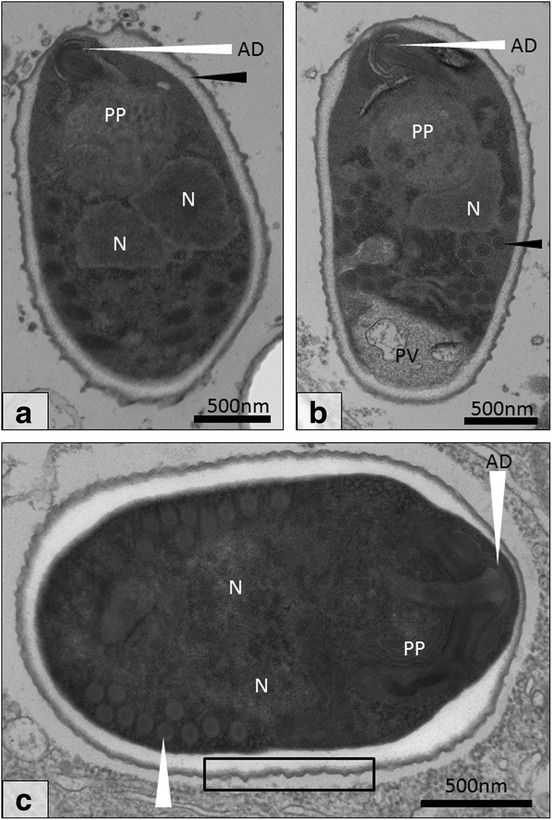
Final development stages of *Cucumispora roeselii* n. sp. **a** Diplokaryotic sporoblast (N) with anchoring disk (AD), polaroplast (PP) and thickened endospore (*black arrow*). **b** A second sporoblast displaying a clear polar vacuole (PV) and polar filament with rings of varying electron density (*black arrow*). **c** The final diplokaryotic (N) spore with bilaminar polaroplast (PP), anchoring disk (AD) and polar filament (9–10 turns; *white arrow*). The spore wall thins at the anchoring disk (AD) whilst being thickest at the periphery of the anchoring disk. Note the ‘thorned’ spore exterior (*black rectangle*). *Scale-bars*: 500 nm

**Fig. 6:**
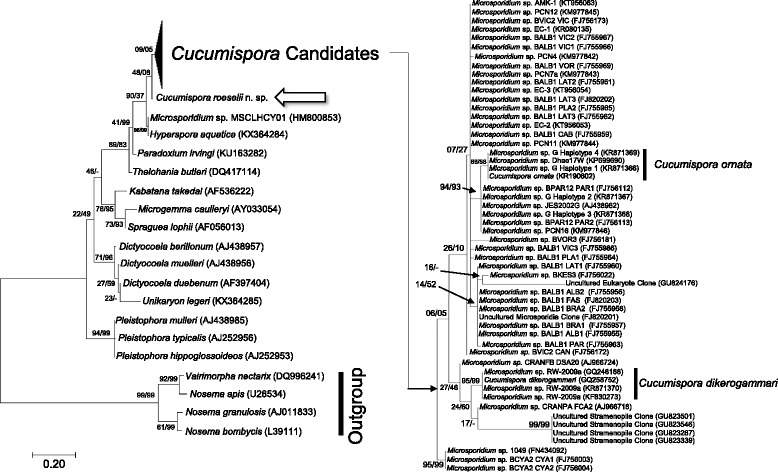
A maximum likelihood tree including the bootstrap confidence for ML/NJ phylogenies. If the neighbour joining phylogeny did not produce a node observed on the maximum likelihood tree, a ‘-’ is noted. The tree is displaying the position of *Cucumispora roeselii* n. sp. (*arrow*), Cucumispora-related SSU isolates (“*Cucumispora* Candidates”), various ‘Clade V’ representatives, and various ‘Clade IV’ representatives (as according to Vossbrinck & Debrunner-Vossbrinck [42] as a fungal outgroup. Sequences considered to belong to existing members of the *Cucumispora* are labelled with the scientific name and indicated by black bars

**Table 2:** Parasites and pathogens associated with *Gammarus roeselii* (*n* = 156) during this study. The prevalence of each pathogen and parasite in the population sampled from Chojna, Poland, is stated alongside the reference image, if available

Parasite group	Species/Disease	Prevalence (%)	Image ref.
Viruses	*Gammarus roeselii Bacilliform Virus*	12.2	Fig. 2
	Putative gut virus	2.7	Fig. 1f
Bacteria	Epibiotic filamentous bacteria	100	Fig. 1b
	Putative rickettsia-like organism	< 1.0	Fig. 1g
Microsporidia	*Cucumispora roeselii* n. sp.	12.2	Figs. 3–5
	*Microsporidium* sp. from the hepatopancreas	< 1.0	Fig. 1j
Protists	Epibiotic, stalked, ciliated protists	83.9	Fig. 1a, b
	Epibiotic embedded ciliated protists	83.9	Fig. 1c
	Parasitic ciliated protists	< 1.0	Fig. 1d
	Gut-dwelling gregarines	50.0	Fig. 1e
Metazoa	Epibiotic rotifer	48.6	Fig. 1a
	Digenean trematodes	1.4	Fig. 1h
	*Polymorphus minutus*	1.4	Fig. 1i
	*Pomphorhynchus* sp.	4.1	No image

The carapace and appendages of *G. roeselii* were often coated with stalked ciliates and epibiotic rotifers (Fig. 1a), however the gills and brood pouch were commonly associated with all epibiotic commensals. None of the epibiotic commensals induced an immune response from the host and were common throughout the *G. roeselii* population (Table 2).

A single animal was observed with a ciliated protist infection in the haemolymph, with accumulations of the parasite in the antennal gland, gills (Fig. 1d), heart and appendages. No immune response toward the parasitic protist was noted throughout the histological screen.

Gregarines (Apicomplexa) were commonly associated with the gut (50% prevalence) (Fig. 1e) and less frequently, the hepatopancreatic tubules (< 1%). Gregarines were often seen in large numbers in the gut with both extracellular and intracellular developmental stages with occasional observation of syzygy. Gregarines elicited no apparent immune response from the host but were detected in significant numbers in the gut lumen.

A putative gut-epithelial virus was observed in 4 individuals where gut nuclei were present with an expanded, eosinophilic viroplasm, resulting in nuclear hypertrophy and marginated host chromatin (Fig. 1f). No immune response was observed against this virus in the histology. In addition, a bacilliform virus was identified from the hepatopancreas of *G. roeselii* and is detailed below.

A putative RLO in the cytoplasm of hepatopancreatocytes was observed in a single individual (Fig. 1g). The cytoplasm of infected cells appeared dense, granular and purple in colour (H&E stain), a common feature of RLO infections in other hosts. Host nuclei were unaffected and no immune responses were observed in affected tissues.

Three metazoan parasites were observed infecting *G. roeselii* (see Table 2 for prevalence details). Digeneans were encysted in the gut, gonad and hepatopancreas (Fig. 1h). Large acanthocephalans such as *Polymorphus minutus* (Fig. 1i) and *Pomphorhynchus* sp*.* were often present in the same tissue types but never together in the same host. No helminth species elicited an immune response from the host.

Two microsporidian infections were observed during screening; the first in the hepatopancreas and the second in the muscle (the muscle infecting microsporidian is detailed below). The microsporidian from the hepatopancreas was observed in a single specimen fixed for histology, meaning that no ethanol or glutaraldehyde fixed materials were taken, resulting in a lack of information for full taxonomic analysis for this species. This microsporidian was present only in the hepatopancreas; specifically, in the cytoplasm of infected cells where several development stages could be seen in histological section (Fig. 1j). No immune response was observed against this microsporidian; however, disintegration of infected tubules was observed.

### *Gammarus roeselii* bacilliform virus: histopathology and TEM

A novel virus infecting the nuclei of hepatopancreatocytes was observed using histology and TEM. Histologically, the virus was present only in the nuclei of infected hepatopancreatocytes and caused host chromatin margination and nuclear hypertrophy due to an expanded viroplasm. Uninfected cell nuclei showed normal chromatin configuration without expanded viroplasm (Fig. 2a inset). This viral pathology was present in 12.2% of specimens (Fig. 2a).

TEM of an infected hepatopancreas tubule and associated cells revealed a viroplasm consisting of large bacilliform virus particles in the host cell nucleus (Fig. 2b). Virions were rod-shaped and consisted of an electron dense, cylindrical core (length 177.4 ± 18 nm, width 35.9 ± 6 nm) and, were surrounded by a single membrane (length 224.0 ± 17 nm, width 70.0 ± 13 nm) (Fig. 2c). Currently no genetic data are available for this virus. This novel virus is termed *Gammarus roeselii* Bacilliform Virus (GrBV) until further data can be acquired, to allow for taxonomic identification.

### Microsporidian histopathology

The microsporidian present in the musculature of *G. roeselii* causes an externally visible opacity in infected amphipod due replacement of muscle fibres with masses of parasites. Histologically, microsporidian spores were seen throughout the musculature of 12.2% of individuals (Fig. 3a), with early-stage infections apparently limited to the muscle fibre periphery (Fig. 3b). No microsporidian spores were observed in other host organs or tissues. Often, melanisation reactions and, haemocyte aggregation were associated with clusters of spores (Fig. 3c) with some evidence of spore phagocytosis by haemocytes. Via histology, mature spores appeared eosinophilic (pink) (Fig. 3a) with earlier developmental stages (e.g. meronts) appearing blue-purple in section (Fig. 3b).

### Microsporidian life-cycle and ultrastructure

Ultrastructurally, the developmental cycle of the microsporidian in *G. roeselii* resembled that of *C. dikerogammari* described by Ovcharenko et al. [23] and *C. ornata* described by Bojko et al. [22]. Infected muscle fibres contained tightly packed merogonial and sporogonial life stages, which developed in direct contact with the host muscle cytoplasm; often in the sarcolemmal space. The microsporidian development began with a diplokaryotic meront (2n) bound by a thin cell membrane (Fig. 4a). Nuclear division of the diplokaryotic meront formed a tetranucleate merogonal plasmodium (2 × 2n) present with a string of four nuclei separated by a thin membrane (Fig. 4b). The tetranucleate meront plasmodium can show early thickening of the cell membrane (Fig. 4b) prior to its division to form two diplokaryotic sporonts (2n), which show further thickening of the cell membrane prior to any formation of spore extrusion apparatus (Fig. 4c-d). Later stage sporonts developed an electron dense cytoplasm prior to formation of early spore extrusion apparatus (Fig. 4e). The maturing sporoblast became electron dense and cucumiform in shape, with an early anchoring disk and coiled, irregular-shaped, polar filament in cross-section (Fig. 4f). The condensed sporoblast displayed the earliest development of an electron lucent endospore (Fig. 4f) and became increasingly turgid during spore maturation (to presume an oval shape) (Fig. 5a-b). Further thickening of the electron-lucent endospore, circularisation of the polar filament cross-sections and development of spore organelles, such as the polaroplast and polar vacuole, occurred in the late sporoblast (Fig. 5a-b). At this stage, the exospores resumed an irregular surface (most clearly seen in the image of the final spore, Fig. 5c).

The final diplokaryotic spore was 2.2 ± 0.1 μm in length (*n* = 30) and 1.5 ± 0.1 μm in width (*n* = 30), contained an anchoring disk, bi-laminar polaroplast, 9–10 turns of the polar filament [cross-sectional diameter: 92 ± 13 nm (*n* = 30)] with rings of proteins at varying electron density, thickened spore wall (plasmalemma, endospore, exospore) and a ribosome-rich, electron-dense cytoplasm (Fig. 5c). The spore wall was of variable thickness according to location; thinnest at the terminal point of the anchoring disk (40 ± 6 nm) and thicker elsewhere (up to 185 ± 50 nm).

### Microsporidian phylogeny

The amplicon derived from the microsporidian infecting the musculature of *G. roeselii* provided an 825 bp sequence of the SSU rRNA gene. This sequence showed closest similarity to *Microsporidium* sp*.* 1049 (FN434092.1: 98% similarity; query cover: 99%; e-value = 0.0) a microsporidian isolated from *Gammarus duebeni duebeni* from Dunstaffnage Castle (Scotland, UK), and *Microsporidium* sp*.* MSCLHCY01 (HM800853.2: 96% similarity; query cover: 96%; e-value = 0.0) a microsporidian isolated from the copepod *Lepeophtheirus hospitalis*, parasitizing the starry flounder, *Platichthys stellatus*, from British Colombia, Canada. The closest named species were *Cucumispora ornata* (KR190602.1: 95% similarity; query cover: 99%; e-value = 0.0), a microsporidian pathogen isolated from the invasive demon shrimp, *Dikerogammarus haemobaphes* Eichwald, from the Carlton Brook invasion site, UK, and *Cucumispora dikerogammari* (GQ246188.1: 93% similarity; query cover: 96%; e-value = 0.0), a microsporidian isolated from the killer shrimp, *Dikerogammarus villosus* Sowinsky, from an invasion site in France. Several microsporidian SSU sequences show high similarity (~90–100%) to those corresponding to the genus *Cucumispora* and are included in Additional file 1: Table S1, depicting their host and geographical origin.

This novel microsporidian sequence branched at the base of the *Cucumispora* with low bootstrap confidence (Fig. 6). The closest phylogenetic associations were with *Microsporidium* sp. 1049, *Microsporidium* sp*.* BCYA2 CYA1 (FJ756003.1: 98% similarity; query cover: 63%; e-value = 0.0) and *Microsporidium* sp*.* BCYA2 CYA2 (FJ756004.1: 98% similarity; query cover: 63%; e-value = 0.0). Each “*Microsporidium* sp.” has no supporting developmental or morphological data. The clade identified as “*Cucumispora* candidates” (highlighted in Fig. 6) is differentiated (bootstrap support = 90–37%) from the closest taxonomically identified genus *Hyperspora* Stentiford, Ramilo, Abollo, Kerr, Bateman, Feist, Bass & Villalba, 2016 (which includes a hyperparasitic microsporidian). Some of the SSU sequences present in the “*Cucumispora* candidates” may be associated with this genus but without developmental or ultrastructural information it is difficult to be sure. The microsporidian sequence isolated by this study is separate from *Microsporidium* sp*.* MSCLHCY01 (an isolate closely associated with *H. aquatica* at 95–99%) on the tree, despite the overall sequence similarity (96%) (Fig. 6).

### Description of a new species of *Cucumispora*

**Order Crustaceacida Stentiford, Bateman, Small, Moss, Shields, Reece & Tuck, 2010**

**Family Myosporidae Stentiford, Bateman, Small, Moss, Shields, Reece & Tuck, 2010**

**Genus** ***Cucumispora*** **Ovcharenko, Bacela, Wilkinson, Ironside, Rigaud & Wattier, 2010**

#### Cucumispora roeselii n. sp.

***Type-host*** **:** *Gammarus roeselii* (Gammaridae) collected from outside its native range.

***Type-locality*** **:** Chojna, (52.966N, 14.42906E), Oder River Basin, Poland.

***Type-material*** **:** Histological sections and TEM resin blocks of the *C. roeselii* n. sp. infected *G. roeselii* tissues are deposited in the Registry of Aquatic Pathology (RAP) at the Cefas Laboratory, Weymouth, UK.

***Site in host*** **:** Infections are restricted to the musculature of *G. roeselii*. Microsporidian spores can be seen in haemocytes likely due to phagocytosis.

***Representative DNA sequence:*** SSU rDNA sequence was deposited in the GenBank database under accession number KY200851.

***ZooBank registration*** **:** To comply with the regulations set out in article 8.5 of the amended 2012 version of the *International Code of Zoological Nomenclature* (ICZN) [32], details of the new species have been submitted to ZooBank. The Life Science Identifier (LSID) of the article is urn:lsid:zoobank.org:pub:EA191185-6A61-4AB5-81B9-AEE9110F881F. The LSID for the new name *Cucumispora roeselii* is urn:lsid:zoobank.org:act:B6BE8D23-8383-4FED-AD1B-259628D064F9.

***Etymology*** **:** The specific epithet “*roeselii*” is derived from the host species, which refers to the thorns down the back of the animal that resemble those of a rose (Rosoideae). It also holds an additional meaning, referring to the “thorned” appearance of the spore wall in this new microsporidian species.

### Description

Ultrastructurally, spores appear oval (length 2.2 ± 0.1 μm; width 1.5 ± 0.1 μm), with a “thorned” spore wall consisting of an electron lucent endospore and electron dense exospore at varying thicknesses either around the spore (138 ± 27 nm), at the point of the anchoring disk (40 ± 6 nm), or at the periphery of the anchoring disk (185 ± 50 nm). The polar filament turns between 9–10 times around the centre and posterior of the spore. This parasite is diplokaryotic throughout its life-cycle. Similarity of the SSU rDNA sequence to the type species *C. dikerogammari* was 93%. Transmission information is currently unavailable but predicted to be horizontal as derived from the pathology; no infection of the gonad was observed.

## Discussion

This study presents the first comprehensive pathogen screen of the non-native gammarid, *G. roeselii*, outside of its native range and includes a taxonomic description of a novel species of microsporidian belonging to the genus *Cucumispora*. The novel microsporidian is named herein as *Cucumispora roeselii* n. sp. Studies such as this one are important to advise risk assessment criteria for invasive and non-native species, specifically in the light of absent information on the pathogens and parasites of invasive and non-native species [2]. While *G. roeselii* has previously been considered as a low-impact invader, in this case we identify *G. roeselii* as a potentially high-profile invader because of its status as a pathogen carrier, transferring pathogens along its route of introduction and spread. It is important to consider if these pathogens could transmit to native wildlife, if they act as a regulator for the host species; limiting its potential impact when present, or if they could be used against the invader in a targeted biological control approach.

### *Cucumispora roeselii* n. sp. and the genus *Cucumispora*

The evidence provided by this study recognises a novel aquatic microsporidian parasite that shows ultrastructural (9–10 turns of polar filament; bi-laminar polaroplast), developmental (diplokaryotic life-cycle), histopathological (muscle-infecting) and genetic (SSU similarity of 93%) similarities to the type-species of *Cucumispora*, *C. dikerogammari* [23].

*Gammarus roeselii* is not of Ponto-Caspian origin or part of the genus *Dikerogammarus*, as the hosts of both previously described *Cucumispora* spp. [22, 23]. *Cucumispora dikerogammari* and *C. ornata* are both thought to originate in the native range of their hosts. However the inclusion of *C. roeselii* n. sp. in this genus requires reconsideration of the origins and range of *Cucumispora* spp. Were this parasite to have originated from the hosts native range (The Balkans) it could indicate an interesting phylogeographic spread of microsporidia within this genus. There is a possibility that this parasite has been acquired from the Polish environment, and/or from other invaders.

Several genetic isolates provide strong sequence similarity to members of the *Cucumispora* [17, 21–23, 33–36], Unpublished works through BLASTn] (Additional file 1: Table S1; Fig. 6). The ranges of these sequenced parasite isolates belong mainly to European territories, but some studies demonstrate isolates from Caribbean and Canadian waters [34, 36]. This information suggests that members of the genus *Cucumispora* may be present around the globe, and their recent identification further suggests their role as emergent pathogens, not only in gammarids but in copepods as well [36]. However, recently published information suggests that hyperparasitic microsporidia with the capability to infect protists appear to have similar SSU sequences to the *Cucumispora* and have been placed into the recently erected genus *Hyperspora* [37]. Until further information is provided in the form of legitimate taxonomic descriptions from more of the SSU isolates in Fig. 6, the native/invasive range and host range of many potential *Cucumispora* spp*.* remains an interesting phenomenon.

Some isolates show close relatedness to taxonomically described *Cucumispora* spp*.* (Fig. 6). *Microsporidium* sp*.* G (haplotypes 1, 2, 3 and 4) isolated from *D. haemobaphes* (Germany) is 99% similar to *Cucumispora ornata* and clades closely in the tree presented in Fig. 6. It is likely these are the same parasite and should be synonymised [17]. However, determining a taxonomic basis on a single gene does not propagate a strong scientific standing and histological and TEM evidence for *Microsporidium* sp*.* G from both *D. haemobaphes* and *G. roeselii* should be confirmed in each host before amalgamating.

### Microbial associations and invasion biology of *Gammarus roeselii*

Several pathogens, parasites and commensals were identified histologically as part of this study. *Polymorphus minutus* and *Pomphorhynchus* sp*.* represent two known acanthocephalan parasites of *G. roeselii* (Table 1) also observed in this sample from Chojna. Epibiotic rotifers, ciliated protists and filamentous bacteria are commonly associated with aquatic species [3, 38] as are gut dwelling gregarines in amphipod hosts [3, 39].

Digenean associations with amphipods are also common and several are known to utilise amphipods as intermediate hosts before entering further hosts where they can reach sexual maturity [40]. Digeneans detected in this study were of an undetermined species (possibly multiple species) and its/their life-cycle and reason for parasitizing *G. roeselii* is currently unknown.

The parasitic ciliated protist (Fig. 1d) has not been noted from *G. roeselii* in the past and is likely a novel association for this species. Without DNA sequence data it is uncertain whether this parasite is taxonomically novel or not. Parasitic ciliates have been noted in amphipods in the past, such as *Fusiforma themisticola* Chantangsi, Lynn, Rueckert, Prokopowicz, Panha & Leander, 2013, which parasitizes *Themisto libellula* (Lichtenstein in Mandt) [41].

A second microsporidian association in this study was of a rare parasite (<1% prevalence) targeting the hepatopancreas of *G. roeselii*. Most microsporidia that target the hepatopancreas of crustaceans fall into the ‘Clade IV’ of microsporidian taxonomy (Terresporidia) [42] and further, into the Hepatosporidae [43, 44]. Obtaining TEM and SSU sequence data would help to taxonomically identify this species. A recent study by Grabner et al. [17] revealed two microsporidian SSU sequences, isolated from *G. roeselii*, that correspond to microsporidia from Group IV (Terresporidia); the histopathology presented by this study may link to one of these isolates and further tests should be carried out to confirm this and identify the species taxonomically.

A single observation of a putative RLO in the cytoplasm of infected hepatopancreatocytes is an interesting association as few RLOs have been noted from amphipods in the past. To date, the only examples include putative *Rickettsiella*-like SSU rDNA sequences available from BLASTn (NCBI) and systemic haemolymph infections caused by RLOs in *Gammarus pulex* (L.) [45] and *Crangonyx floridanus* Bousfield [46].

### Viruses in the Amphipoda

A variety of viruses have been identified from Crustacea either morphologically, via DNA sequence data or through searching for endogenous viral elements in the genome of crustacean hosts [47–49]. Few have ever been identified from hosts belonging to the Order Amphipoda. To date only three published viral associations have been made from amphipods: the first is in the form of histology and TEM images of a bacilliform virus from the hepatopancreas of *Dikerogammarus villosus* and referred to as *Dikerogammarus villosus Bacilliform Virus* (DvBV) [3]; the second, an unassigned circovirus from a *Gammarus* sp. [50]; and the third includes various circular-virus associations to *Diporeia* spp. [51].

Although DvBV was, previous to this study, the only visually confirmed virus from an amphipod, bacilliform viruses from the hepatopancreas of crustaceans are common and several have been identified morphologically (Table 3). GrBV, isolated from the hepatopancreas of *G. roeselii* in this study, fits morphologically and pathologically alongside the viruses in Table 3. *Penaeus monodon nudivirus* (PmNV) has been the focus of genome sequencing efforts, revealing that this group of morphologically-similar viruses are likely nudiviruses (*Nudiviridae*) [52]. Further genome sequencing and generalised primer-designs for nudivirus genes would benefit this area greatly and allow further taxonomic insight into the viral life history.

**Table 3:** Bacilliform viruses from the hepatopancreas of several Crustacea

Organism	Host species	Bacilliform virus from the HP	Reference
Crayfish	*Astacus astacus*	AaBV	[56]
	*Cherax quadricarinatus*	CqBV	[57]
	*Pacifasticus leniusculus*	PlBV	[58]
	*Cherax destructor*	CdBV	[59]
	*Austropotamobius pallipes*	ApBV	[60]
Crab	*Cancer pagurus*	CpBV	[61]
	*Carcinus maenas*	CmBV	[38]
	*Pinnotheres pisum*	PpBV	[62]
Shrimp	*Crangon crangon*	CcBV	[63]
	*Penaeus monodon*	PmNV	[52]
Amphipod	*Dikerogammarus villosus*	DvBV	[3]
	*Gammarus roeselii*	GrBV	Present study

The viral pathology in the gut of *G. roeselii* remains putative due to a lack of appropriately fixed material to observe virions via TEM. Pathologically, the presence of the infection (nuclei of gut epithelia) suggests a DNA virus. It is uncertain at this point whether this infection is caused by GrBV simply infecting a separate tissue type; this cannot be diagnosed using our current data and materials. Re-sampling and TEM processing should provide informative data, however genetic data would be most beneficial; a valid point for many of the viruses in Table 3.

### *Cucumispora roeselii* n. sp.: invasion threat or beneficial for control?

Although the prospect of invaders carrying pathogens pose a potential problem [1, 53], in some instances parasites can act as controlling agents [54]. This phenomenon may be taking place with the *D. haemobaphes* invasion of the UK, where the microsporidian pathogen, *C. ornata,* may be limiting the health of the invasive population [22]. Amphipod populations without their microsporidian pathogens are not regulated as they would be in their native range, and loss of their “enemies” may result in greater fitness and a higher impact on the environment; such as that observed with the killer shrimp at invasion sites in the UK [3, 55].

*Gammarus roeselii* is considered to be a low impact non-native species [12] in freshwater systems across Europe [6, 8–10, 12]. However, this non-native host may not be the main issue but instead its pathogens could act as “biological weapons” to facilitate invasion and harm wildlife [1, 2, 53]. The concept of being a pathogen carrier is often ignored in risk assessment, often due to a lack of information around the capability to accurately assess the risk invasive pathogens pose [2]. Possible parasite transmission from *G. roeselii* to native fauna is high; this is based on the large diversity of parasites and pathogens observed by this study. Due to limited records, it is difficult to be certain which pathogens and parasites are from the native range of *G. roeselii* and which have been acquired during its introduction and spread. Assessment of co-evolved pathogens in the native range of *G. roeselii* would increase our understanding of the origins of *C. roeselii* n. sp. and the other pathogens observed during this study. Examples of enemy release in gammarids are available, including: the loss of pathogens during the introduction process [3] and of gammarids carrying pathogens into novel invasion sites [22, 35].

It may be possible that the pathogens identified as part of this study regulate the host species, and escape from these regulators could increase the impact and risk of *G. roeselii*. Understanding the associated mortality rate, host range, behavioural alterations and physiological changes these pathogens impose upon their host would allow further assessment of whether these pathogens are regulating non-native *G. roeselii* populations in Chojna and elsewhere within Europe. Information gleaned from such studies could define whether *C. roeselii* n. sp., and other pathogens associated with *G. roeselii*, could be useful as biocontrol agents, or if they are emerging diseases and detrimental for vulnerable wildlife.

## Conclusions

This study has identified several pathogens and parasites, which utilise *G. roeselii* as their host; including a novel species description of a microsporidian parasite. These pathogens could pose a significant threat to native wildlife. This example study displays the importance of screening non-native, low impact invaders for pathogens to identify their potential to carry and transmit wildlife disease to native fauna and flora. Disease profiling should be factored into the risk assessment of invasive and non-native species and current assessment should not rely on host-focussed studies alone.

## Additional file

Additional file 1: Table S1. Geographical and host data for those microsporidian gene isolates that clade within the “*Cucumispora* candidates” group in Fig. 6. (DOCX 20 kb)

## Abbreviations

NNS: Non-native species
SSU: Small subunit ribosomal RNA
