# Supplementary material for: Parasites, pathogens and commensals in the “low-impact” non-native amphipod host Gammarus roeselii
Source: Parasit Vectors. 2017 Apr 20;10:193. doi: 10.1186/s13071-017-2108-6 (PMC5397875; doi:10.1186/s13071-017-2108-6)
Supplement: Additional file 1: Table S1. — Geographical and host data for those microsporidian gene isolates that clade within the “Cucumispora candidates” group in Fig. 6. (DOCX 20 kb) [file 13071_2017_2108_MOESM1_ESM.docx]

**Additional file 1: Table S1** Geographical and host data for those microsporidian gene isolates that clade within the “*Cucumispora* candidates” group, in Fig. 6.

| **Microsporidian SSU isolate** | **Host** | **Geographical location** | **Hosts invasive or native range** | **Reference** |
| --- | --- | --- | --- | --- |
| *Microsporidium* sp. BALB1 PLA1 | *Micruropus platycercus* | Russia: Lake Baikal | Native range | Unpublished |
| *Microsporidium* sp. BALB1 VIC2 | *Acanthogammarus victorii* | Russia: Lake Baikal | Native range | Unpublished |
| Uncultured Microsporidia clone BALB1 LAT3 | *Gmelinoides fasciata* | Russia: Lake Baikal | Native range | Unpublished |
| *Microsporidium* sp. BALB1 PLA2 | *Micruropus platycercus* | Russia: Lake Baikal | Native range | Unpublished |
| *Microsporidium* sp. BALB1 LAT3 | *Brandtia latissima latior* | Russia: Lake Baikal | Native range | Unpublished |
| *Microsporidium* sp. BALB1 CAB | *Garjajewia cabanisii* | Russia: Lake Baikal | Native range | Unpublished |
| *Microsporidium* sp. PCN11 | *Pallasea cancellus* | Russia: Lake Baikal | Native range | [1] |
| *Microsporidia* sp. EC-1 | *Eulimnogammarus cyaneus* | Russia: Lake Baikal | Native range | Unpublished |
| *Microsporidium* sp. PCN4 | *Pallasea cancellus* | Russia: Lake Baikal | Native range | [1] |
| *Microsporidium* sp. PCN7a | *Pallasea cancellus* | Russia: Lake Baikal | Native range | [1] |
| *Microsporidium* sp. PCN12 | *Pallasea cancellus* | Russia: Lake Baikal | Native range | [1] |
| *Microsporidium* sp. BALB1 VOR | *Linevichella vortex* | Russia: Lake Baikal | Native range | Unpublished |
| *Microsporidium* sp. BALB1 LAT2 | *Brachyuropus grewingkii* | Russia: Lake Baikal | Native range | Unpublished |
| *Microsporidium* sp. BVOR3 | *Linevichella vortex* | Russia: Lake Baikal | Native range | Unpublished |
| *Microsporidium* sp. BALB1 VIC1 | *Acanthogammarus victorii* | Russia: Lake Baikal | Native range | Unpublished |
| *Microsporidium* sp. BALB1 BRA1 | *Macrohectopus branickii* | Russia: Lake Baikal | Native range | Unpublished |
| *Microsporidium* sp. BALB1 BRA2 | *Macrohectopus branickii* | Russia: Lake Baikal | Native range | Unpublished |
| *Microsporidium* sp. BKES3 | *Pallaseopsis kessleri* | Russia: Lake Baikal | Native range | Unpublished |
| Uncultured Microsporidia clone BALB1 FAS | *Gmelinoides fasciata* | Russia: Lake Baikal | Native range | Unpublished |
| *Microsporidium* sp. BALB1 PAR | *Dorogostaiskia parasitica* | Russia: Lake Baikal | Native range | Unpublished |
| *Microsporidium* sp. BALB1 ALB2 | *Ommatogammarus albinus* | Russia: Lake Baikal | Native range | Unpublished |
| *Microsporidium* sp. BALB1 ALB1 | *Ommatogammarus albinus* | Russia: Lake Baikal | Native range | Unpublished |
| *Microsporidium* sp. BALB1 LAT1 | *Brandtia latissima latior* | Russia: Lake Baikal | Native range | Unpublished |
| *Microsporidium* sp. BVIC2 CAN | *Pallasea cancellus* | Russia: Lake Baikal | Native range | Unpublished |
| *Microsporidium* sp. BVIC2 VIC | *Acanthogammarus victorii* | Russia: Lake Baikal | Native range | Unpublished |
| *Microsporidium* sp. G (Dh4-6) | *Dikerogammarus haemobaphes* | Germany | Invasive range | [2] |
| *Microsporidium* sp. G (Dh2-10) | *Dikerogammarus haemobaphes* | Germany | Invasive range | [2] |
| *Microsporidium* sp. G (Dh2-3) | *Dikerogammarus haemobaphes* | Germany | Invasive range | [2] |
| *Cucumispora ornata* | *Dikerogammarus haemobaphes* | UK: River Trent | Invasive range | [3] |
| *Microsporidium* sp. PCN16 | *Pallasea cancellus* | Russia: Lake Baikal | Native range | [1] |
| *Microsporidium* sp. BPAR12 PAR1 | *Dorogostaiskia parasitica* | Russia: Lake Baikal | Native range | Unpublished |
| *Microsporidium* sp. BPAR12 PAR2 | *Dorogostaiskia parasitica* | Russia: Lake Baikal | Native range | Unpublished |
| *Microsporidium* sp. G (Gr2-10) | *Gammarus roeselii* | Germany | Invasive range | [2] |
| *Microsporidium* sp. G (Gr2-12) | *Gammarus roeselii* | Germany | Invasive range | [2] |
| *Microsporidium* sp. JES2002G | *Gammarus chevreuxi* | UK: River Avon | Native range | [4] |
| Uncultured Microsporidia clone BFAS11 | *Gmelinoides fasciata* | Russia: Lake Baikal | Native range | Unpublished |
| *Microsporidium* sp. BCYA2 CYA1 | *Eulimnogammarus cyaneus* | Russia: Lake Baikal | Native range | Unpublished |
| *Microsporidium* sp. 1049 | *Gammarus duebeni duebeni* | UK: Scotland | Native range | [5] |
| *Microsporidium* sp. BCYA2 CYA2 | *Eulimnogammarus cyaneus* | Russia: Lake Baikal | Native range | Unpublished |
| *Cucumispora roeselii n. sp.* | *Gammarus roeselii* | Poland: Chonja | Invasive range | This Study |
| *Microsporidium* sp. CRANFB | *Crangonyx floridanus* | USA: Florida: River Styx | Native range | [6] |
| *Microsporidium* sp. CRANPA | *Crangonyx pseudogracilis* | France: Cellettes: Beuvron | Invasive range | [6] |
| *Cucumispora dikerogammari* = *Microsporidia* sp. RW-2009a | *Dikerogammarus villosus* | France | Invasive range | [7] |
| *Cucumispora dikerogammari* = *Microsporidia* sp. RW-2009a | *Dikerogammarus villosus* | Poland | Invasive range | [7] |
| *Microsporidium* sp. RW-2009a haplotype 1 | *Dikerogammarus villosus* | Germany | Invasive range | [2] |
| Uncultured Stramenopile clone AB3F14RJ14H09 | Water sample | Cariaco Basin, Caribbean Sea | N/A | [8] |
| Uncultured Stramenopile clone AB3F14RJ2E02 | Water sample | Cariaco Basin, Caribbean Sea | N/A | [8] |
| Uncultured Stramenopile clone AB3F14RJ145F08 | Water sample | Cariaco Basin, Caribbean Sea | N/A | [8] |
| Uncultured Stramenopile clone AB3F14RJ3B02 | Water sample | Cariaco Basin, Caribbean Sea | N/A | [8] |
| Uncultured Stramenopile clone AB3F14RJ7B02 | Water sample | Cariaco Basin, Caribbean Sea | N/A | [8] |

**References**

1. Madyarova EV, Adelshin RV, Dimova MD, Axenov-Gribanov DV, Lubyaga YA, Timofeyev MA. Microsporidian parasites found in the hemolymph of four baikalian endemic amphipods. PloS one. 2015;10(6):e0130311.
2. Grabner DS, Weigand AM, Leese F, et al. Invaders, natives and their enemies: distribution patterns of amphipods and their microsporidian parasites in the Ruhr Metropolis, Germany. Parasites and vectors*.* 2015;8(1):1-15.
3. Bojko J, Dunn AM, Stebbing PD, et al. *Cucumispora ornata* n. sp. (Fungi: Microsporidia) infecting invasive ‘demon shrimp’ (*Dikerogammarus haemobaphes*) in the United Kingdom. Journal of invertebrate pathology. 2015;128:22-30.
4. 21 Terry RS, Smith JE, Sharpe RG, et al. Widespread vertical transmission and associated host sex–ratio distortion within the eukaryotic phylum Microspora. *Pr*oceedings of the Royal Society of London B: Biological Sciences. 2004;271(1550):1783-1789.
5. Krebes L, Blank M, Frankowski J, et al. Molecular characterisation of the Microsporidia of the amphipod Gammarus duebeni across its natural range revealed hidden diversity, wide-ranging prevalence and potential for co-evolution. Infection, Genetics and Evolution. 2010;10(7):1027-1038.
6. Galbreath JGS, Smith JE, Becnel JJ, Butlin RK, Dunn AM. Reduction in post-invasion genetic diversity in *Crangonyx pseudogracilis* (Amphipoda: Crustacea): a genetic bottleneck or the work of hitchhiking vertically transmitted microparasites?. Biological Invasions. 2010;12(1):191-209.
7. Ovcharenko MO, Bacela K, Wilkinson T, et al. *Cucumispora dikerogammari* n. gen. (Fungi: Microsporidia) infecting the invasive amphipod *Dikerogammarus villosus*: a potential emerging disease in European rivers. Parasitology. 2010;137(2):191-204.
8. Orsi W, Edgcomb V, Jeon S, et al. Protistan microbial observatory in the Cariaco Basin, Caribbean. II. Habitat specialization. The ISME journal. 2011;5(8):1357-1373.
